# Pilot assessment of vascular endothelial growth factor receptors and trafficking pathways in recurrent and metastatic canine subcutaneous mast cell tumours

**DOI:** 10.1002/vms3.66

**Published:** 2017-06-30

**Authors:** Lucas Da Silva, Carlos E. Fonseca‐Alves, Jennifer J. Thompson, Robert A. Foster, Geoffrey A. Wood, Renee L. Amorim, Brenda L. Coomber

**Affiliations:** ^1^ Department of Biomedical Sciences University of Guelph Guelph Ontario Canada; ^2^ Department of Veterinary Clinic University of São Paulo State ‐UNESP Botucatu São Paulo Brazil; ^3^ Department of Pathobiology Ontario Veterinary College University of Guelph Guelph Ontario Canada

**Keywords:** VEGFR2, Neuropilin‐1, c‐CBL, dog

## Abstract

Canine subcutaneous mast cell tumour (scMCT) shows less aggressive biological behaviour than cutaneous MCT. Vascular endothelial growth factor receptor 2 (VEGFR2) is expressed by neoplastic cells in canine scMCT, but the relevance of this signalling pathway for disease pathobiology is not clear. The objective of this study was to quantify VEGF‐A, VEGFR2, pVEGFR2, the VEGF co‐receptor Neuropilin 1 (NRP‐1) and the E3 ubiquitin protein ligase c‐Cbl in canine scMCT, and to evaluate their association with disease outcome. Immunohistochemical staining for biomarkers was quantified from 14 cases of canine scMCT using manual and computer‐assisted methods. Kaplan–Meier curves were generated for disease‐free survival (DFS) and compared using Mantel–Cox log‐rank analysis. Cases with high levels of neoplastic cell VEGFR2, pVEGFR2 or c‐CBL immunoreactivity had significantly reduced DFS. All cases displayed neoplastic cells positive for VEGF‐A, which was significantly associated with pVEGFR2 immunoreactivity. There were also significant positive correlations between VEGFR2 and pVEGFR2, and between c‐CBL and pVEGFR2 levels. This pilot study demonstrates the potential utility of these markers in a subset of scMCT in dogs.

## Introduction

Mast cell tumours (MCTs), account for 7–21% of all canine skin cancer (Welle *et al*. 2008) and there are subcutaneous and dermal (also called cutaneous) types. The events resulting in MCT development are unknown, but breed predisposition is well documented and may indicate an underlying genetic component (Warland & Dobson 2013). Activation of the receptor tyrosine kinase KIT plays a role in normal mast cell growth and development, as well as in malignant transformation (London *et al*. 1999; Webster *et al*. 2007; Warland & Dobson 2013). Studies with the *c‐KIT* gene have indicated a key role in dermal MCT development and progression (London *et al*. 1999; Jones *et al*. 2004; Webster *et al*. 2007; Warland & Dobson 2013; Patruno *et al*. 2014). The KIT protein, encoded by the proto‐oncogene *c‐KIT*, stimulates the proliferation, maturation and activation of normal mast cells (Yavuz *et al*. 2002). Its role in subcutaneous MCT is less well understood (Thompson *et al*. 2011a, 2016).

Toceranib and masitinib are receptor tyrosine kinase inhibitors specifically designed to treat canine MCT and represent the first targeted therapies for canine cancer patients (London 2013). Toceranib targets KIT, VEGFR, PDGFR and Flt‐3, while masitinib targets KIT and PDGFR (London 2009). As these tyrosine kinase inhibitors have multiple targets, interpretation of their activity in a clinical setting is challenging. For instance, masitinib significantly improved long‐term survival in dogs with non‐resectable MCTs, regardless of KIT mutation status (Hahn *et al*. 2010). Up to 60% of dogs with MCT given a grade of III do not have detectable KIT mutations and no KIT mutations have been reported in subcutaneous MCT (Hahn *et al*. 2010; Thompson *et al*. 2011a). Given the complex interplay between signalling pathways in these neoplastic cells, further investigation of other potential prognostic and therapeutic targets would be informative.

Vascular endothelial growth factor (VEGF) is an important regulator of endothelial cell proliferation, vasculogenesis, angiogenesis and vascular permeability, mediated primarily by the tyrosine kinase receptor VEGFR2 (Ferrara *et al*. 2003; Folkman 2006). Canine MCT, both dermal and subcutaneous, are reported to express VEGFR2 (Rebuzzi *et al*. 2007; Thompson *et al*. 2016) and mast cell tumour cells express both VEGF ligand and VEGFRs (Sekis *et al*. 2009) although whether autocrine signalling pathways are active in mast cell tumours is controversial. Neuropilin‐1 (NRP‐1) is a VEGF co‐receptor expressed on endothelial cells and some neoplastic cells, acting to modulate VEGF signalling by regulating VEGFR2 recycling, at least in endothelial cells (Soker *et al*. 1998, 2002; Kawasaki *et al*. 1999; Hong *et al*. 2007; Adham *et al*. 2010; Zhang & Simons 2014).

Casitas B‐lineage lymphoma proto‐oncogene (*Cbl*) proteins are a highly conserved family of E3 ubiquitin ligases which mono‐ubiquitinate their targets KIT, VEGFRs and PDGFRs (along with other receptor molecules), routing them for destruction via multivesicular bodies/lysosomes (Masson *et al*. 2006; Ryan *et al*. 2006; Swaminathan & Tsygankov 2006; Meyer *et al*. 2011). Loss of c‐CBL led to development of mastocytosis in transgenic mice associated with defective ubiquitination and subsequent accumulation of KIT and disrupted signalling (Bandi *et al*. 2009; Orinska *et al*. 2010). KIT expression and signalling was also found in human acute myeloid leukaemia cells with *c‐Cbl* mutations (Makishima *et al*. 2012).

Subcutaneous MCT develops in the subcutaneous region, and in most cases, is well defined and delimited by fatty tissue (Newman *et al*. 2007). While the majority of canine subcutaneous MCT are low grade and biologically benign, some show local recurrence and metastasis (Newman *et al*. 2007; Thompson *et al*. 2011a,b; Risselada *et al*. 2015). Due to the limited information on prognostic factors in canine subcutaneous MCT, and the potential for RTK inhibition as a therapeutic strategy, this research aims to evaluate the prognostic significance of VEGFR2, NRP‐1 and c‐CBL protein markers in a subset of canine subcutaneous MCT.

## Materials and methods

### Case selection and case characteristics

Paraffin blocks from 14 canine subcutaneous MCTs were used for this pilot study. There were 11 female spayed and three male neutered dogs in this study. The age at diagnosis ranged from 5 to 13 years, with a median age of 7 years. There were 13 purebred dogs (six Labrador Retrievers and seven other purebred dogs) and one mixed breed dog. Neoplastic cells were within 1 mm of the surgical margins in each tumour. Three dogs developed metastasis to lymph node and all dogs eventually developed local recurrence at the original surgical site. No dogs received chemotherapy and one dog received radiation for recurrent disease.

### Western blotting

We performed western blotting analysis to validate the cross reactivity of the primary antibodies, using lysates from the MCT‐1 cell line developed in our laboratory from a grade III MCT derived from a 7‐year‐old male castrated Shar‐pei dog. The blots were blocked with 3% bovine serum albumin in TBS‐T (10 mmol/L Tris–HCl pH 7.5, 150 mmol/L NaCl, 0.1% Tween‐20) for 2 h and incubated overnight with the following primary antibodies: mouse monoclonal anti‐*α*‐tubulin (diluted 1:400 000; Sigma‐Aldrich, Oakville, ON, Canada), rabbit monoclonal anti‐VEGFR2 (1:2000), anti‐phospho‐VEGFR2 (1:2000) or anti‐NRP‐1 (1:1000), or rabbit polyclonal anti‐c‐CBL (1:1000); all from Cell Signaling Technology, Danvers, MA, USA. Membranes were washed three times for 5–10 min in TBS‐T and incubated in HRP‐labelled goat polyclonal anti‐mouse or anti‐rabbit secondary antibodies (both at 1:20 000; Sigma‐Aldrich) for 1 h. Membranes were incubated with chemiluminescent HRP substrate Luminata™ Forte (EMD Millipore, Darmstadt, Germany) and bands were visualized in a Bio‐Rad ChemiDocTM XRS+ system (Bio‐Rad Laboratories Canada Inc., Mississauga, ON, Canada).

### Histology and immunohistochemistry

Paraffin sections (5 *μ*m thickness) were placed on Superfrost+ slides for histology and immunohistochemistry (IHC). Slides were deparaffinized in xylene and subsequently rehydrated in a graded isopropanol series. For immunohistochemistry, antigen retrieval was performed in sodium citrate buffer (pH 6.0) in a 95°C water bath for 15 min. Endogenous peroxidase activity was quenched with 3% hydrogen peroxide for 30 min and non‐specific binding sites were blocked with a 1:1 ratio of 5% normal goat serum (Life Technologies, Burlington, ON, Canada) and DAKO block (Dako, Carpinteria, CA, USA) for 2 h at room temperature followed by overnight incubation at 4°C with the anti‐VEGFR2 (1:1600); anti‐phospho‐VEGFR2 (1:125); anti‐NRP‐1 (1:11 600) or anti‐c‐CBL (1:200), followed by washing and incubation in biotinylated anti‐rabbit IgG (1:200; Vector Laboratories, Burlington, ON, Canada) for 1 h, at room temperature. Peroxidase‐streptavidin complex (1:200; Cedarlane, Burlington, ON, Canada) was then applied for 1 h at room temperature, and peroxidase activity visualized with 3,3′‐diaminobenzidine tetrahydrochloride (DAB; Vector Laboratories). Negative controls consisted of slides incubated with no primary antibody and slides incubated with an irrelevant primary antibody (rabbit polyclonal anti‐pan‐cytokeratin; Fig. S1). Positive controls consisted of vascular endothelial cells for VEGFR2, pVEGFR2 and NRP‐1 (internal control) and canine testis for c‐CBL (Fig. S1). Slides were counterstained with Harris haematoxylin, dehydrated and coverslipped. Immunohistochemical labelling for VEGF‐A was conducted by the Michigan State University Diagnostic Center for Animal and Population Health (Lansing, MI, USA). Additional slides were stained with haematoxylin and eosin, and used to assess mitotic count, numbers of multinucleate cells and tumour growth pattern as previously reported (Thompson *et al*. 2011b).

### Image analysis

The slides were viewed using a compound light microscope (DMLB100 T, Leica Microsystems, Wetzlar, Germany) and five images were captured from each section with QCapture software (QImaging, Surrey, BC, Canada) at high‐power (40X objective) fields. Representative areas were systematically selected for immunostaining analysis, avoiding regions of necrosis, edges of sections and regions lacking neoplastic cells. The images were captured and analysed in a blinded fashion, and clinical information was not available until after image analysis was completed. Staining was evaluated by establishing a ‘threshold’ using Image J software (ImageJ v1.47; NIH, Bethesda, MD, USA), and determining the percent positively stained area for each image. The threshold was established for each case based on staining of positive control cells. Reaction product associated with vascular endothelium was subtracted from images immunostained for VEGFR2, pVEGFR2 or NRP‐1 as appropriate. The percent positive area score for each case was calculated by taking the average of all five fields, and the median positive area was determined for all 14 cases. Based on that median, cases were divided into high expression tumours and low expression tumours and the outcome was compared between both groups. For VEGF‐A immunoreactivity, slides were scored in a blinded fashion using a modification of published methods (Rebuzzi *et al*. 2007): +++, > 90% neoplastic cells strongly reactive; ++, > 90% neoplastic cells positive; +, > 50% neoplastic cells positive; +/−, < 50% neoplastic cells positive.

### Statistical analysis

Statistical analysis was performed using GraphPad Prism (version 7) (GraphPad, La Jolla, CA, USA). Kaplan–Meier curves were generated and Mantel–Cox log‐rank analysis was used to analyse disease‐free survival (DFS) defined as interval between diagnosis and detection of disease progression (local recurrence and/or metastasis). Linear regression analysis was performed to compare quantification for VEGFR2 and pVEGFR2, and for c‐CBL and pVEGFR2 immunoreactivity, and differences in pVEGFR2 expression according to VEGF expression were compared by Mann–Whitney test for unpaired analysis. The cut off points for mitotic count and multinucleate cells were established as previously reported (Thompson *et al*. 2011b). For all analysis, a value of 95% (*P* ≤ 0.05) was used for statistical significance.

## Results

**Figure 1:**
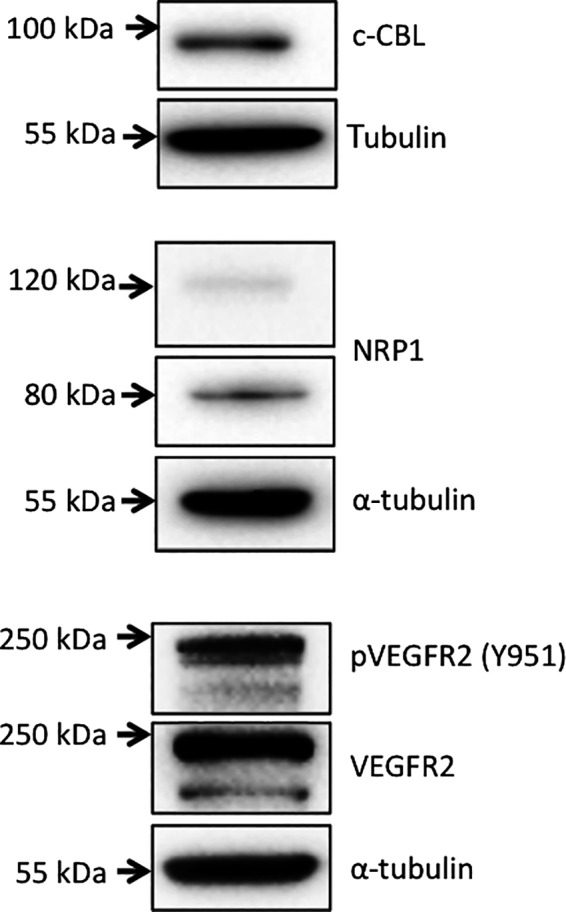
Western blot detection of proteins of interest in lysates of canine MCT cell line MCT‐1; detection of *α*‐tubulin was used to control for protein loading.

Western blotting analysis was performed to validate the cross reactivity of the primary antibodies with canine proteins (Fig. 1). VEGFR2 and pVEGFR2 antibodies identified a major band at approximately 250 kDa, and minor bands of slightly lower molecular weight, as has been reported for human and canine neoplastic cells (Adham & Coomber 2009; Thompson *et al*. 2016). The c‐CBL blots showed a single specific band of approximately 100 kDa (Feshchenko *et al*. 1998), while NPR‐1 blots demonstrated full length and truncated isoforms, as has been reported for human NRP‐1 (Gagnon *et al*. 2000). The same antibodies were then employed for immunolocalization of antigens in FFPE samples of canine subcutaneous MCT.

**Figure 2:**
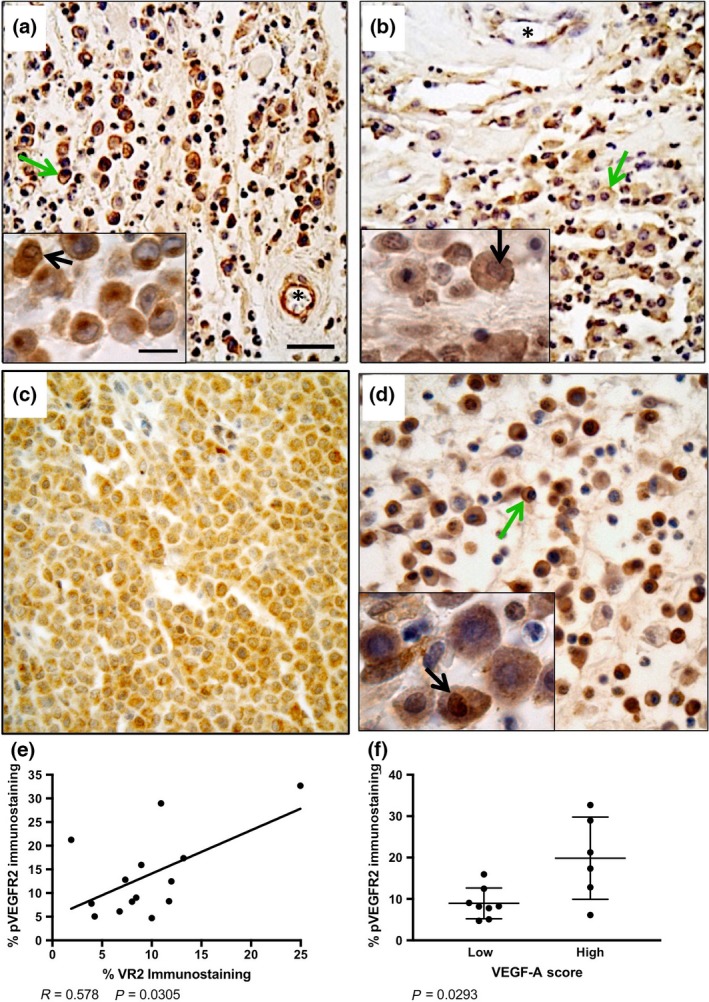
Immunohistochemical detection of VEGFR2 (a), pVEGFR2 (b), VEGF‐A (c) and NRP‐1 (d) in canine subcutaneous mast cell tumours. Blood vessels positive for vascular endothelial staining of VEGFR2 and pVEGFR2 are indicated by asterisks. Nuclear immunoreactivity is indicated by black arrows and membranous immunoreactivity is indicated by green arrows. Scale bar = 50 *μ*m. (e) Linear regression analysis of canine subcutaneous mast cell tumours reveals a significant correlation in percent positive pixels for native VEGFR2 and pVEGFR2. (f) Cases with higher VEGF‐A scores (++ and +++) had significantly more pVEGFR2 immunoreactivity. VEGFR, Vascular endothelial growth factor receptor 2; NRP‐1, Neuropilin 1.

**Figure 3:**
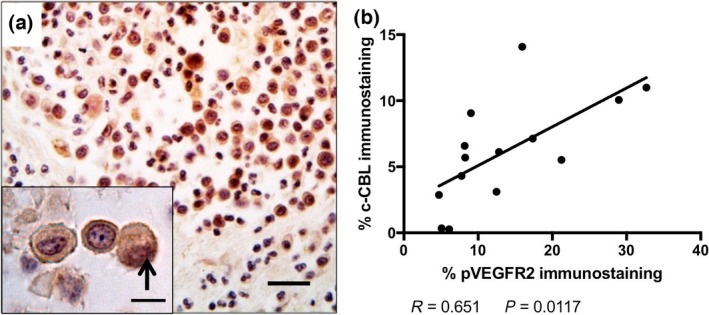
(a) Immunohistochemical detection of c‐CBL in canine subcutaneous mast cell tumours. Nuclear immunoreactivity is indicated by the black arrow. Scale bar = 50 *μ*m. (b) Linear regression analysis of canine subcutaneous mast cell tumours reveals a significant correlation in percent positive pixels for pVEGFR2 and c‐CBL. VEGFR, Vascular endothelial growth factor receptor 2.

The immunohistochemical protocols developed were successfully used on all 14 MCT samples with minimal background staining (Figs. 2, 3). Negative controls involved replacing the primary antibody with PBS, and use of an irrelevant immunogen (pan‐cytokeratin) (Fig. S1). VEGFR2 and pVEGFR2 immunostaining of neoplastic MCT cells was both membranous (green arrows) and cytoplasmic, and vascular endothelial cells in blood vessels (asterisks) were strongly positive (Fig. 2a,b). Positive nuclear immunolocalization of neoplastic cells was also occasionally seen (black arrows). VEGF immunolocalization was predominantly cytosolic (Fig. 2c). The VEGF co‐receptor NRP‐1 showed predominantly cytoplasmic with some membranous immunostaining in neoplastic cells (green arrow); nuclear immunolocalization was also occasionally present (black arrow, Fig. 2d). Comparison of manual counting (number of positive cells/field) vs. Image J analysis revealed a significant positive correlation (*P* = 0.0014; Fig. S2), thus we proceeded to use the higher throughput image analysis. Quantification of IHC staining revealed a significant positive correlation between neoplastic cell VEGFR2 and pVEGFR2 immunoreactivity (*P* = 0.0305; Fig. 2e). VEGF scores were grouped into ‘low’ (+/− and +) and ‘high’ (++ and +++), and pVEGFR2 expression levels were compared for each group. Cases with high VEGF scores had significantly higher pVEGFR2 levels (*P* = 0.0293; Fig. 2f).

Scatter plots showing range of positive immunoreactivity scores for VEGFR2, pVEGFR2, NRP‐1 and c‐CBL are shown in Figure S3. Cases were stratified into ‘Low’ and ‘High’ based on median values for percent positive neoplastic cell pixel immunoreactivity. Low expression of VEGFR2 or pVEGFR2 by MCT neoplastic cells was significantly associated with increased DFS (*P* = 0.0476 and *P* = 0.0289, respectively; Table 1). There were no statistical differences between NRP‐1 expression and DFS (Table 1). The E3 ubiquitin ligase enzyme c‐CBL showed diffuse to intense cytoplasmic immunolocalization, with occasional nuclear staining (arrows) in MCT neoplastic cells (Fig. 3a). Low expression of c‐CBL by MCT neoplastic cells was significantly associated with increased DFS (*P* = 0.0029; Table 1). There was also a significant positive correlation between neoplastic pVEGFR2 and c‐CBL immunolocalization (*P* = 0.0117; Fig. 3b). Significant differences in DFS of this case set were not seen with tumour growth pattern or presence of multinucleated cells (Table 1). Mitotic count >4 in 10 hpf was significantly associated with reduced DFS (*P* = 0.0100; Table 1), as has been reported Thompson *et al*. (2011b), but outcome stratified by c‐CBL staining achieved higher statistical significance and displayed greater Hazard Ratios (Table 1).

**Table 1:** Disease‐free survival by tumour parameters

Parameter	Hazard ratio^a^	Median DFS (days)	Probability^b^
NRP‐1			
Low	1.61	179	0.8809
High	0.6211	292.5	
VEGFR2			
Low	0.3908	364.5	0.0476
High	2.559	105	
pVEGFR2			
Low	0.3750	399	0.0289
High	2.667	73	
c‐CBL			
Low	0.2776	409	0.0029
High	3.602	73	
Mitotic index^c^			
≤4	0.3093	404	0.0100
>4	3.233	105	
Multinucleation^d^			
Yes	1.378	137	0.5125
No	0.7258	399	
Growth pattern			
Infiltrative	2.22	303	0.1863
Other^e^	0.4504	186	

a By log‐rank analysis.

b By log‐rank (Mantel–Cox) test.

c Number of mitoses per 10 high‐power (400X) fields.

d Presence or absence of at least 1 mutinucleated cell per 10 high‐power (400 X) fields.

e Other growth patterns include nodular and circumscribed.

## Discussion

Here, we report that a subset of recurrent canine subcutaneous MCTs were heterogeneous in their expression of VEGFR2, with membranous, cytoplasmic, or nuclear immunolocalization in neoplastic cells. In parallel, we detected phosphorylated (activated) VEGFR2 in similar cellular compartments. In vascular endothelial cells, membrane‐associated VEGFR2 undergoes tyrosine phosphorylation upon ligand binding, leading to the activation of signalling pathways that affect the proliferation, survival, permeability and migration of the cell (Domingues *et al*. 2011). Nuclear localization of pVEGFR2 in endothelial cells regulates its own transcription and potentially increases the angiogenic response (Domingues *et al*. 2011). Cytosolic localization of VEGFR2 may be indicative of high levels of VEGFR2 stored in intracellular vesicles, awaiting signalling to direct its trafficking to the plasma membrane (Manickam *et al*. 2011). Neoplastic cells from a variety of cancer types use VEGFR2 as an autocrine growth factor, and may employ a similar process to that observed in endothelial cells (von Marschall *et al*. 2000; Strizzi *et al*. 2001; Sher *et al*. 2009; Adamcic *et al*. 2012; Darrington *et al*. 2012). Levels of neoplastic cell VEGFR2 expression were positively associated with lymph node status and a trend towards shorter survival time in canine mammary carcinoma (Diessler *et al*. 2016). Here, we found a significant positive correlation between neoplastic cell expression of VEGFR2 and of pVEGFR2. Since positive VEGF‐A immunoreactivity was seen in all cases, it suggests a possible role for VEGFR autocrine signalling in the pathobiology of canine subcutaneous MCT. Our results are also in line with a recent study that reported VEGFR2 expression (detected via western blot) in neoplastic cells isolated from some canine cutaneous and subcutaneous MCT (Thompson *et al*. 2016). These findings may help explain the success of KIT directed TKIs in tumours that lack mutated KIT (London *et al*. 2009).

Recently, alterations in the *KDR* (VEGFR2) gene have been reported for some human cancers. Over one‐third of human malignant melanomas are reported to contain a Q472H germline variation, located in the fifth IgG‐like repeat (Silva *et al*. 2016). BLAST alignment of the human VEGFR2 amino acid sequence with the putative canine version [EMBL nucleotide sequence database #AAEX03009081] shows 93% homology, with the relevant glutamine residue conserved between human and canine. Amplification of the *KDR* gene (≥4 copies) is also reported in human non‐small cell lung cancer (Nilsson *et al*. 2016). Cancer cell lines displaying *KDR* copy number gain showed enhanced VEGFR2 protein levels and activation of alternative signalling pathways compared to NSCLC cells lacking copy number amplification. The KDR mutation analysis and copy number status and their relationship with VEGFR expression in canine cutaneous MCTs is worth evaluating in future studies.

Neuropilins (NRPs) belong to the large group of axon guidance molecules, playing key roles in migration of neural crest cells and axonal processes during development. In addition to their involvement as co‐receptors for angiogenic signalling via VEGF, NRPs are also implicated in human cancer progression, including leukaemia (Grandclement & Borg 2011). High NRP‐1 expression by neoplastic cells was associated with lymph node metastasis and poor prognosis in human oral squamous cell carcinoma (Chu *et al*. 2014). Overexpression of NRP‐1 induced enhanced invasiveness in malignant melanoma cells (Bai *et al*. 2015) and induction of epithelial to mesenchymal transition (EMT) and cancer stem cell phenotype in oral squamous cell carcinoma (Chu *et al*. 2014). The group of MCT studied expressed NPR‐1, but this did not correlate with disease‐free survival, suggesting this is not a good prognostic factor for subcutaneous MCT.

c‐CBL mutations are found in human leukaemia where they are implicated in disrupted KIT signalling (Tefferi 2010; Makishima *et al*. 2012; Schwaab *et al*. 2012; Traina *et al*. 2012). Acting as a tumour suppressor, loss of *CBL* expression could confer a more aggressive phenotype; yet, in our study, we found high c‐CBL levels were associated with worse DFS. CBL proteins can also be involved as adaptor molecules independent of their E3 ligase activity, leading to activation of PI3K, AKT and MAPK and cytoskeletal pathways, among others (Swaminathan & Tsygankov 2006; Liyasova *et al*. 2015). While most CBL mutations lead to loss of E3 ligase activity, such mutant proteins can maintain their signal‐enhancing functions (Schwaab *et al*. 2012). In support of this, we found a significant positive correlation between levels of neoplastic cell pVEGFR2 and c‐CBL in these canine subcutaneous mast cell tumours. Interestingly, it was recently reported that high levels of c‐CBL expression were significantly associated with poor outcome in human glioma (Jing *et al*. 2016).

Canine subcutaneous mast cell tumours are distinct from dermal lesions in both their location and biological behaviour. The prognosis of subcutaneous MCT can be better determined using recognized histological parameters such as mitotic count, Ki67 and AgNOR score and immunohistochemical KIT expression pattern (Thompson *et al*. 2011a). In this pilot study, we extend the evaluation of canine subcutaneous mast cell tumours and determine that VEGFR2 and c‐CBL expression could further stratify aggressive lesions for improved prognosis. The study is limited by the small sample size and retrospective nature of the case selection. However, our findings support exploration of VEGFR as a potential target in recurrent or metastatic subcutaneous MCTs. Future approaches could potentially include use of current KIT targeted inhibitors that may have anti‐VEGFR activity, and/or therapies that directly target VEGFR signalling. The impact of the biomarkers evaluated here on disease pathobiology, and their potential utility in less aggressive subcutaneous mast cell tumours or for cutaneous mast cell tumours, is currently under investigation.

## Source of funding

This study was supported by grants from the Ontario Veterinary College Pet Trust Fund (#051425) and the São Paulo Research Foundation (FAPESP #2014/25583‐1).

## Conflict of interest

The authors declare that they have no conflicts of interest.

## Ethics statement

The authors confirm that the ethical policies of the journal, as noted on the journal's author guidelines page, have been adhered to and the appropriate ethical review committee approval has been received. The Canadian Council for Animal Care (CCAC) guidelines, under the supervision of the University of Guelph local Animal Care Committee were followed.

## Contributions

LDS, CEF‐A & JJT performed the western blotting, immunohistochemistry and data analysis. JJT, GAW, FAF and RLA provided clinical and pathological expertise in data analysis. BLC conceived this study and provided data analysis. All authors contributed to the writing and revising of this work.

## Supporting information


**Figure S1.** Control procedures for immunohistochemistry.

**Figure S2.** Regression analysis illustrating the correlation between scoring.
**Figure S3.** Scatter plots of Mean Pixel area for parameters evaluated.
