# Supplementary figures and images for: Pilot assessment of vascular endothelial growth factor receptors and trafficking pathways in recurrent and metastatic canine subcutaneous mast cell tumours
Source: Vet Med Sci. 2017 Jun 30;3(3):146–55. doi: 10.1002/vms3.66 (PMC5645839; doi:10.1002/vms3.66)

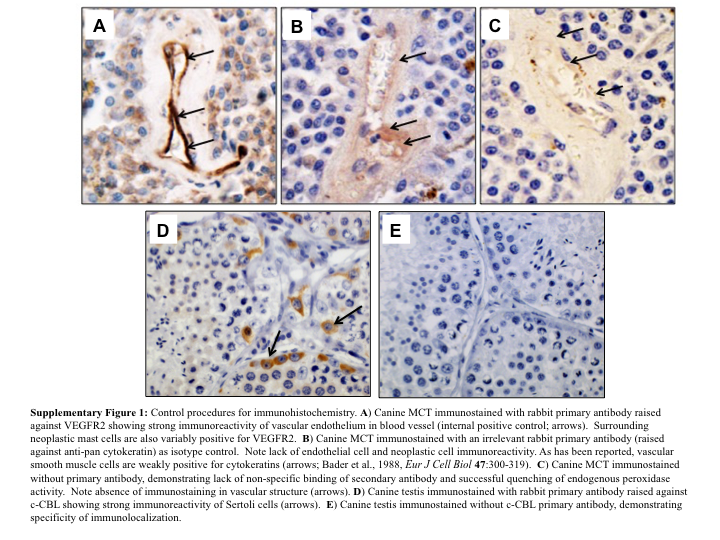

Supplement: Supplementary file 1 — Figure S1. Control procedures for immunohistochemistry. [file VMS3-3-146-s001.tiff]

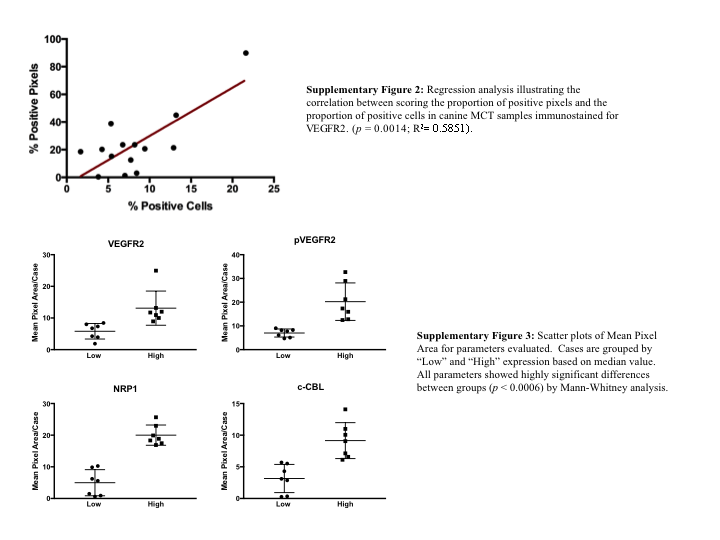

Supplement: Supplementary file 2 — Figure S2. Regression analysis illustrating the correlation between scoring. Figure S3. Scatter plots of Mean Pixel area for parameters evaluated. [file VMS3-3-146-s002.tiff]
